# Detection of *Sarcocystis hominis*, *Sarcocystis bovifelis*, *Sarcocystis cruzi*, *Sarcocystis hirsuta* and *Sarcocystis sigmoideus* sp. nov. in carcasses affected by bovine eosinophilic myositis

**DOI:** 10.1016/j.fawpar.2024.e00220

**Published:** 2024-01-19

**Authors:** Selene Rubiola, Gastón Moré, Tiziana Civera, Andrew Hemphill, Caroline F. Frey, Walter Basso, Irene Colasanto, Davide Vercellino, Marta Fidelio, Mauro Lovisone, Francesco Chiesa

**Affiliations:** a Department of Veterinary Sciences, University of Turin, 10095 Grugliasco, TO, Italy; b Institute of Parasitology, University of Bern, 3012 Bern, Switzerland; c ASL TO3 di Collegno e Pinerolo, SC Igiene degli Allevamenti e delle Produzioni Zootecniche, 10093 Collegno, TO, Italy; d ASL di Asti, Servizio Veterinario Area B, 14100 Asti, AT, Italy

**Keywords:** *Sarcocystis* spp., Bovine eosinophilic myositis, Cattle, *Sarcocystis hominis*, *Sarcocystis sigmoideus* sp. nov

## Abstract

Bovine eosinophilic myositis is an inflammatory myopathy characterized by multiple focal or diffuse grey to green patches leading to condemnation of affected carcasses. Although its etiology is still uncertain, there is evidence that *Sarcocystis* species may play a role in the development of eosinophilic myositis. The goal of the present study was to identify *Sarcocystis* spp. in intralesional and extralesional tissues of condemned cattle carcasses, in order to evaluate the possible role of different bovine *Sarcocystis* spp. in the etiology of bovine eosinophilic myositis. Muscle samples (*n* = 100) of 26 affected carcasses were collected in Northern Italy. One to five samples with lesions and two aliquots of tissue without lesions were collected from each carcass; lesions were grossly categorized in green focal lesions and green diffuse patches. Genomic DNA was extracted and analyzed by multiplex-PCR targeting different *Sarcocystis* spp. Unidentified species were characterized morphologically (light microscopy, histology), ultrastructurally (scanning and transmission electron microscopy) and on the molecular level (complete 18S rRNA gene and partial *cox1* gene sequencing). A bovine eosinophilic myositis prevalence of 0.017% was visually assessed by routine carcass inspection between 2014 and 2019 in Italy (184/1,108,150 slaughtered cattle). Out of 26 carcasses, 25 revealed the presence of at least one *Sarcocystis* species (96.2%). The presence of *Sarcocystis* spp. DNA was significantly more frequent in intralesional than in extralesional samples. Considering the different species, *Sarcocystis bovifelis* and *Sarcocystis hominis* were significantly more frequent in intralesional (41.7% and 50%, respectively) than in extralesional samples (1.9% and 15.4%, respectively), while there was no significant difference between the presence of *Sarcocystis cruzi* and *Sarcocystis hirsuta* in intralesional (27.1% and 2.1%, respectively) and extralesional (30.8% and 1.9%, respectively) samples. The presence of an unnamed *Sarcocystis* sp. showing thick-walled (3.7–5.4 μm) cysts with densely packed, flattened, undulating and narrow protrusions, which showed an S-shape in side view, was recorded in the diaphragm of two carcasses. Genomic DNA from individual sarcocysts isolated from the diaphragm was successfully amplified and further sequenced. Sequence comparison revealed <94.6% and 83.4% identity at 18S rRNA and *cox1* genes, respectively, with other named *Sarcocystis* spp., while the phylogenetic analysis clearly separated the unnamed *Sarcocystis* sp. from the other *Sarcocystis* spp. using cattle as intermediate hosts. The present study contributes to the understanding of the importance of different *Sarcocystis* spp. in the pathogenesis of bovine eosinophilic myositis. The results emphasize the association of *Sarcocystis hominis* and *Sarcocystis bovifelis* with bovine eosinophilic myositis and highlight the presence of a new *Sarcocystis* sp. using cattle as intermediate hosts. The name *Sarcocystis sigmoideus* sp. nov. is proposed for the newly described *Sarcocystis* species.

## Highlights

• A bovine eosinophilic myositis prevalence of 0.017% was recorded in Northern Italy.
• Carcasses affected by bovine eosinophilic myositis were analyzed by multiplex-PCR.
• *Sarcocystis* spp. DNA was more frequently detected in intralesional samples.
• *S. hominis* and *S. bovifelis* DNA was more frequently detected in intralesional samples.
• *Sarcocystis sigmoideus* sp. nov. using cattle as intermediate hosts is described.

## Graphical abstract

**Figure:**
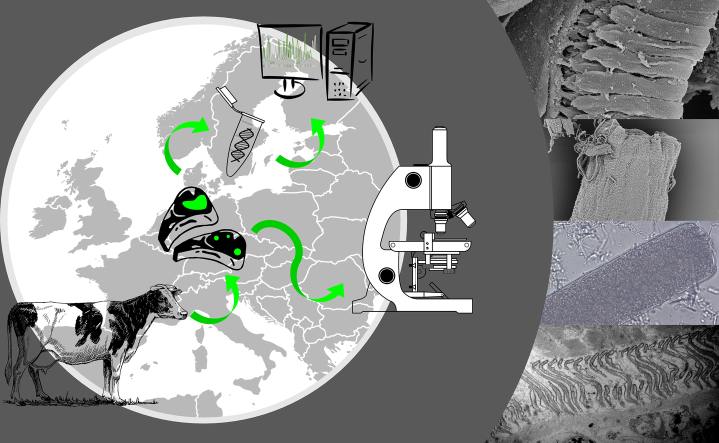

## 1 Introduction

Bovine eosinophilic myositis (BEM) is a rare subclinical disease affecting cattle (*Bos taurus*), typically detected during meat inspection in slaughterhouses and meat cutting plants. Gross lesions are usually described as multiple grey-yellow-green to white round or fusiform focal patches or stripes observed in skeletal and cardiac muscles (Dubey and Rosenthal, 2023; Wouda et al., 2006); a less common but more evident presentation of BEM includes diffuse grey-green patches, which can involve larger portions of muscle tissues. The most frequently affected areas are masticatory muscles, tongue, heart, and diaphragm, although in severe cases all striated muscles can be involved. The histopathological alterations consist of an eosinophilic inflammation between muscle fibers leading to myocyte degeneration and the formation of granulomas with a central area of necrosis (Vangeel et al., 2013). The green color results from the accumulation of eosinophil granulocytes, whereas the yellow color is caused by the degeneration of tissues and the white color by fibrosis (Dubey and Rosenthal, 2023). BEM is usually subclinical, except for some sporadic reports of acute or chronic clinical disease (Aráoz et al., 2019; Dubey et al., 2016), still the resulting condemnation of the BEM-affected muscles or of the entire carcass leads to considerable economic losses in the beef sector (Commission Implementing Regulation (EU) 2019/627, 2019).

Although the etiology of BEM is still not completely clear, there is evidence that *Sarcocystis* species may play a role in the development of this inflammatory myopathy (Dubey and Rosenthal, 2023; Gajadhar et al., 1987; Rubiola et al., 2021; Vangeel et al., 2013; Vangeel et al., 2012; Wouda et al., 2006). *Sarcocystis* spp. are intracellular protozoan parasites belonging to the phylum Apicomplexa with a worldwide distribution and a two-host life cycle, involving herbivores, omnivores or carnivores as intermediate hosts and carnivores, scavengers, or omnivores as definitive hosts (Dubey et al., 2016). More than 200 species affecting mammals (including humans), reptiles, birds and possibly fishes are described within the genus *Sarcocystis*; among them, seven named species affect cattle as intermediate host: *Sarcocystis hominis* and *S. heydorni,* using humans and non-human primates as definitive hosts, *S. cruzi,* using canids as definitive hosts (Dubey et al., 2023), and *S. hirsuta*, *S. bovifelis*, *S. bovini* and *S. rommeli,* either confirmed or predicted to use felids as definitive hosts, while *S. bovifelis* DNA has also been detected in the intestine of mustelids (Dubey et al., 2015; Dubey, 2022; Gjerde, 2016b; Prakas et al., 2021). In this context, there is uncertainty concerning the validity of *S. rommeli*, which might be a synonym of *S. bovifelis* or *S. bovini* (Dubey and Rosenthal, 2023), while further unnamed species have been molecularly detected in Belgium and Italy (Domenis et al., 2011; Rubiola et al., 2021; Vangeel et al., 2013; Zeng et al., 2021). The prevalence of *Sarcocystis* spp. in cattle can reach up to 100%, with significant variations depending on the country and on the *Sarcocystis* species considered. Based on the recenyliterature, the European prevalence of *S. cruzi* in clinically healthy cattle ranges between 56.5% and 96% and is followed by the prevalence of *S. bovifelis* (8.7–71.6%), *S. hominis* (8.5–21%), *S. hirsuta* (0.9–30.4%), *S. bovini* (2%) and *S. heydorni* (0.5–0.9%) (Dubey and Rosenthal, 2023; Hoeve-Bakker et al., 2019; Hornok et al., 2015; Prakas et al., 2020; Rubiola et al., 2021; Zeng et al., 2021). On the other hand, the prevalence of BEM appears to be quite low, ranging from 0.002% to 5% worldwide, though available data are scarce and outdated (Bradley and Taylor, 1993; Jensen et al., 1986; Vangeel et al., 2013; Fortier et al., 1993). The high prevalence of *Sarcocystis* spp. in cattle might appear inconsistent with the low prevalence of BEM, thereby questioning the possible association or causality between eosinophilic myositis and *Sarcocystis* species. As a possible explanation, the hypothesis of the association of BEM with the occurrence or co-occurrence of specific *Sarcocystis* spp. has been advanced (Rubiola et al., 2021; Vangeel et al., 2013). Reports attesting the presence of thin-walled and thick-walled sarcocysts inside or around BEM lesions can be found in the literature (Dini et al., 2023; Gajadhar et al., 1987; Gajadhar and Marquardt, 1992; Jensen et al., 1986; Rubiola et al., 2021; Vangeel et al., 2013; Vangeel et al., 2007). The most recent articles investigating the association of BEM and *Sarcocystis* spp. have been conducted in Europe. In 2013, Vangeel et al. (2013) detected different *Sarcocystis* spp. in lesions collected from BEM condemned carcasses in Belgium, the majority of which were identified as *S. hominis*; Rubiola et al. (2021) recorded a significantly higher presence of *S. hominis* and *S. bovifelis* in BEM-condemned carcasses than in randomly sampled bovine carcasses in Italy in 2021. Both Vangeel et al. (2013) and Rubiola et al. (2021) also recorded the presence of *S. cruzi*, *S. hirsuta* and an unidentified *Sarcocystis* sp. A recent report by Dini et al. (Dini et al., 2023) described the presence of *S. hominis* and *Toxoplasma gondii* in a BEM condemned beef cattle in Italy. The possible association of the zoonotic *S. hominis* with eosinophilic myositis in cattle further increases the interest around this myopathy, together with the economic impact due to carcass condemnation.

The identification of *Sarcocystis* spp. within BEM lesions is complicated by the degeneration of the sarcocysts that may be present and by the possible co-occurrence of different species within and outside the lesions. Furthermore, misidentifications have occurred in past years due to the use of identification methods based on morphological techniques or molecular markers which couldn't differentiate closely related *Sarcocystis* spp. (Moré et al., 2013; Robertson et al., 2019). The inclusion of new *Sarcocystis* spp. within the group of the well-known *S. hominis*, *S. cruzi* and *S. hirsuta* during the last decade, and the above-mentioned debate concerning the validity of some of the newly named *Sarcocystis* spp., have further complicated the present scenario.

As previously mentioned, Rubiola et al. (2021) investigated and compared the molecular prevalence of *Sarcocystis* spp. in cattle carcasses randomly sampled at slaughter and in BEM-affected carcasses in 2021, highlighting a higher presence of *S. hominis* and *S. bovifelis* in carcasses condemned due to eosinophilic myositis. Nevertheless, the possible presence of different *Sarcocystis* spp. inside and outside lesions of BEM-affected carcasses was not investigated. Therefore, the aim of the present study was to identify *Sarcocystis* spp. in intralesional and extralesional tissues of BEM-condemned cattle carcasses, in order to evaluate the possible role of different bovine *Sarcocystis* spp. in the etiology of eosinophilic myositis.

## 2 Materials and methods

### 2.1 Sample collection

Between January 2019 and November 2022, muscle samples of 26 BEM-condemned carcasses were collected by Official Veterinarians of different slaughterhouses located in Northern Italy during post-mortem inspections of slaughtered animals. Tissue samples, including cardiac and skeletal muscle, were stored at 4 °C and transported to the Laboratory of Food Inspection of the Department of Veterinary Sciences (University of Turin, IT). Upon arrival, muscle samples were macroscopically examined and gross lesions were categorized into two groups: 1- greenish focal lesions (GFL), defined as small (0,5–3 cm) focal, green, yellow-green or grey-white lesions often characterized by a mineralized central core, and 2- greenish diffused patches (GDP), defined as vast areas (up to 15 cm) of green, yellow-green or grey-white muscle in which a central core of fibrosis and mineralization could not be identified. One to five samples with lesions and two aliquots of 25 mg tissue sample without lesions were collected from each carcass. All collected samples were stored at −20 °C until further analysis; the remaining muscle tissue was preserved at −20 °C when available.

### 2.2 Molecular detection of *Sarcocystis* species by multiplex-PCR

Genomic DNA was extracted directly from the collected muscle samples applying the DNeasy Blood and Tissue Kit (QIAGEN, Hilden, Germany), following the manufacturer's tissue protocol; the lysis step was carried out at 56 °C overnight and DNA samples were eluted in 200 μl of elution buffer and kept frozen at −20 °C. The detection and differentiation of *Sarcocystis* spp. was performed applying the multiplex-PCR described by Rubiola et al. (2020) targeting the nuclear small subunit (18S) rDNA and the cytochrome C oxidase subunit I mitochondrial (mtDNA *cox1*) genes of *S. hominis*, *S. cruzi*, *S. hirsuta*, *S. bovifelis* and *Sarcocystis* spp. Extracted DNA from negative cattle muscles as well as reagent blanks as negative controls and positive *Sarcocystis* spp. controls were included in each PCR run. The PCR amplicons were separated in 2% agarose gels stained with GelRed™ Nucleic Acid Gel Stain (Sigma-Aldrich, Germany) and observed in a UV transilluminator (GelDoc XR Imaging System, BioRad, Hercules, CA, USA). Samples revealing only the presence of the *Sarcocystis* spp. fragment (200–250 bp) were considered unidentified and sequenced to achieve species identification. DNA samples of GFL which could be misdiagnosed as degenerated cysticerci (*n* = 41) were tested for the presence of *Taenia saginata* DNA by PCR targeting a fragment of the mtDNA *cox1* gene as described by Chiesa et al. (2010).

### 2.3 Sanger sequencing

PCR amplicons generated by the genus-specific primer set of the multiplex-PCR with no species-specific fragments were purified using ExoSAP-IT™ Express (ThermoFisher Scientific, USA); sequencing reactions were performed applying the ABI Prism BigDye Terminator Cycle Sequencing Ready Reaction Kit, version 1.1 (Applied Biosystems, Foster City, CA). The sequencing products were purified by DyeEX (QIAGEN, Hilden, Germany), and sequence analysis was performed on an Applied Biosystems SeqStudio Genetic Analyzer (Thermo Fisher Scientific, Waltham, MA). The nucleotide sequences were analyzed using the BLASTN sequence similarity search against the NCBI NT database (Altschul et al., 1990).

### 2.4 Morphological and molecular characterization of *Sarcocystis sigmoideus* sp. nov.

#### 2.4.1 Isolation of sarcocysts and morphological characterization

Frozen muscle samples revealing unidentified *Sarcocystis* spp. after sequence analysis of the *Sarcocystis* genus-specific fragments were recovered and thawed at 4 °C in order to proceed with the isolation of single sarcocysts applying the protocol described by Moré et al., with slight modifications (Moré et al., 2013). Briefly, 5–10 g of pooled muscle samples were placed in a 50 ml tube and minced with up to 50 ml of sterile phosphate-buffered saline (PBS) using a tissue homogenizer, filtered with a sieve, and centrifuged at 600 x*g* for 5 min. The supernatant was discarded and the resulting pellet was resuspended in 20 ml of PBS and observed using an inverted microscope at 40× magnification (Stemi 508, Zeiss). Observed cysts or cyst portions were isolated, photographed at 200× and 400× magnification (Nikon Eclipse *Ci* with a calibrated Nikon Camera model DFK 23UP031) and individually stored at − 20 °C for subsequent molecular characterization. A subset of the isolated sarcocysts was collected and fixed in 2.5% glutaraldehyde and 2% osmium tetroxide for transmission electron microscopy (TEM) and scanning electron microscopy (SEM) (Hemphill and Croft, 1997). They were then embedded in Epon 812-resin and viewed on a Morgagni transmission electron microscope operating at 80 kV or coated with gold and observed in a GeminiSEM 450 (ZEISS, Germany), both associated with the Microscopy Imaging Center (MIC) of the University of Bern. Cyst wall ultrastructure was evaluated as previously described (Moré et al., 2013).

Concurrently, BEM lesions were individually dissected under a stereomicroscope with the help of preparation needles; the calcified cores of the granulomatous BEM lesions were isolated from the surrounding muscle fibers and individually stored at −20 °C for molecular characterization, whilst muscle sample portions were fixed in 10% formalin and processed using conventional histology methods. Tissue sections, 4 μm thick, were stained with hematoxylin and eosin (H&E) and observed using a light microscope (Nikon Eclipse Ci). Digital images of sarcocysts on histological sections were acquired while width and wall thickness of the cysts were measured using the Nikon NIS software and recorded.

#### 2.4.2 Molecular characterization and phylogenetic analysis

Single-cyst DNA was extracted using the DNeasy Blood & Tissue Kit (QIAGEN, Hilden, Germany) as described above. Samples were subsequently molecularly characterized amplifying the partial mtDNA *cox1* gene with primers SF1 (Gjerde, 2013) and SR9 (Gjerde, 2014a) targeting a fragment of around 1100 bp as described by Gjerde (2016b) and the complete 18S rRNA gene using the primer sets ERIB1 - S2 (Barta et al., 1997; Fischer and Odening, 1998) and S3 - Primer BSarc (Fischer and Odening, 1998; Gjerde, 2014b) as described by Gjerde (2016b). Extracted DNA from negative cattle muscles as well as reagent blanks as negative controls and a positive *Sarcocystis* spp. control were included in each PCR run. The amplified PCR products were separated in 2% agarose gel stained with GelRed™ Nucleic Acid Gel Stain (Sigma-Aldrich, Germany) and observed in a UV transilluminator (GelDoc XR Imaging System, BioRad, Hercules, CA, USA). Each reaction mixture included 2.5 μl of template DNA. When muscle samples showing the presence of unidentified *Sarcocystis* spp. could not be retrieved to perform cysts isolation and single-cyst DNA extraction, the mixed DNA samples initially tested by multiplex-PCR were used to perform the above-mentioned molecular characterization. The center of BEM lesions (previously dissected and isolated under the stereomicroscope) were subjected to DNA extraction and *cox1* mtDNA amplification following the same protocol mentioned above.

PCR products were purified (ExoSAP-IT, Thermo Fisher Scientific, USA) and sequenced as previously described. Forward and reverse sequences, as well as the 18S rRNA overlapping sequences, were manually assembled into consensus sequences using the Alignment Explorer within MEGA X (Kumar et al., 2016). Primer sequences were removed, and the nucleotide sequences were analyzed using the BLASTN sequence similarity search against the NCBI NT database. Phylogenetic analyses of the mtDNA *cox1* gene and the 18S rRNA gene sequences were conducted separately. For the mtDNA *cox1* gene, 52 partial sequences from 37 taxa were used in the analysis, including 13 new sequences generated in the present study; regarding the 18S rRNA gene, a total of 42 sequences from 38 taxa were used in the analysis, including 5 new sequences generated in the present study. Multiple alignments of the generated sequences and *Sarcocystis* spp. sequences retrieved from GenBank were obtained by using the ClustalW algorithm in MEGA X. Sequences were slightly truncated at both ends, so that all sequences started and ended at the same nucleotide positions. The phylogenetic trees were reconstructed using the neighbour joining method within MEGA X. In both analyses of the mtDNA *cox1* gene and the 18S rRNA gene, *Toxoplasma gondii* was used as outgroup species to root the trees. The phylogeny was tested with the bootstrap method (1000 replicates). The accession numbers of the *Sarcocystis* spp. sequences included in the phylogenetic analyses are shown within the trees.

### 2.5 Statistical analysis

Fisher's exact test was used to compare the proportions and 95% confidence intervals (CIs) of different *Sarcocystis* spp. inside and outside focal and diffuse lesions of BEM-affected carcasses. *P* < 0.05 was considered significant.

## 3 Results

The 26 BEM-condemned carcasses originated from 13 different Provinces of Northern Italy, including Asti, Cuneo and Turin (Piedmont region), Brescia, Cremona, Lodi, Mantua, Milan and Sondrio (Lombardy region), Treviso and Vicenza (Veneto region), Parma (Emilia-Romagna region) and Pordenone (Friuli-Venezia Giulia region). Data related to BEM-condemnation between January 2014 and December 2019 in the biggest slaughterhouse of Northern Italy showed the presence of lesions grossly classified as eosinophilic myositis in 184 out of 1,108,150 slaughtered cattle, resulting in a BEM prevalence of 0.017% (CI95%: 0.014–0.019).

**Fig. 1:**
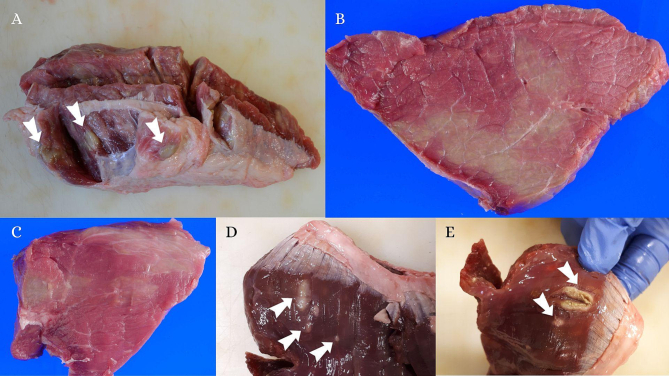
Greenish focal lesions (A, D and E) and greenish diffuse patches (B and C) observed in cattle carcasses affected by lesions grossly classified as eosinophilic myositis. White arrows point out focal lesions detected in skeletal muscles. The color of the lesions ranged from green to grey or white. The green color faded, turning to grey-whitish after air exposure. (For interpretation of the references to color in this figure legend, the reader is referred to the web version of this article.)

Most of the sampled lesions were categorized as GFL (*n* = 41 lesions from 22 carcasses), while 7 lesions (*n* = 7 lesions from 5 carcasses) were described as GDP (Fig. 1). In one out of 26 carcasses both types of lesions were observed. In total, 100 muscle samples were collected, including 52 extralesional (two aliquots per BEM-condemned carcass) and 48 intralesional (one to five samples per BEM-condemned carcass) tissue samples.

All the processed samples tested negative for the presence of *T. saginata* DNA.

### 3.1 Molecular identification of *Sarcocystis* spp. in intralesional and extralesional tissue of BEM-condemned carcasses

Out of 26 BEM-condemned carcasses, 25 carcasses revealed the presence of at least one *Sarcocystis* spp. (96.2%; CI95%: 80.4–99.9).

**Fig. 2:**
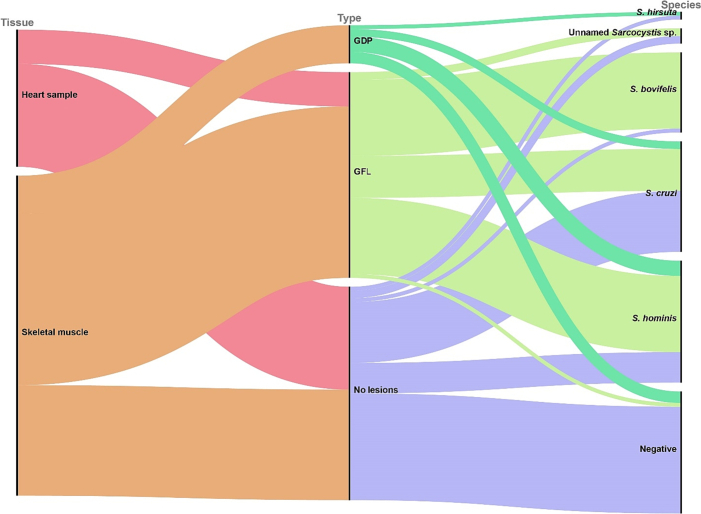
Alluvial diagram showing the distribution of the different *Sarcocystis* species per tissue (heart and skeletal muscle) and per sample type [intralesional - greenish diffuse patches (GDP) and greenish focal lesion (GFL); extralesional]. Rose red: heart; orange: skeletal muscle; bright green: greenish diffuse patches; light green: greenish focal lesions; violet: extralesional muscle. (For interpretation of the references to color in this figure legend, the reader is referred to the web version of this article.)

Considering intralesional samples (*n* = 48), including GDP and GFL, *Sarcocystis* DNA was detected in 91.7% of the samples (44/48; CI95%: 80.0–97.7). *Sarcocystis hominis* was the most common species (50%; CI95%: 35.2–64.8), followed by *S. bovifelis* (41.7%; CI95%: 27.6–56.8), *S. cruzi* (27.1%; CI95%: 15.3–41.9), an unnamed *Sarcocystis* sp. (4.2%; CI95%: 0.5–14.3) and *S. hirsuta* (2.1%; CI95%: 0.1–11.8) (Fig. 1). Mixed infections were observed in 25% (*n* = 12) of the intralesional samples, revealing the presence of up to three *Sarcocystis* spp. at once. When two *Sarcocystis* spp. were detected in the same sample (*n* = 8), the simultaneous presence of *S. hominis* and *S. bovifelis* was the most common finding (n = 4), followed by co-infection of *S. hominis* and *S. cruzi* (*n* = 2), *S. bovifelis* and *S. cruzi* (n = 1), *S. cruzi* and the unnamed *Sarcocystis* sp. (n = 1). In 4 muscle samples the simultaneous presence of three *Sarcocystis* spp. was detected, that is *S. hominis*, *S. bovifelis* and *S. cruzi* (*n* = 3) or *S. hominis*, *S. cruzi* and *S. hirsuta* (n = 1). Taking into account the different lesion type (GFL and GDP), *S. hominis* (20/41, 48.8%; CI95%: 32.9–64.9) and *S. bovifelis* (20/41, 48.8%; CI95%: 32.9–64.9) were the most commonly detected species in GFL, followed by *S. cruzi* (11/41, 26.8%; CI95%: 14.2–42.9), and the unnamed *Sarcocystis* sp. (2/41, 4.9%; CI95%: 0.6–16.5), while *S. hominis* was the most common species in GDP (4/7, 57.1%; CI95%: 18.4–90.1), followed by *S. cruzi* (2/7, 28.6%; CI95%: 3.7–70.9) and *S. hirsuta* (1/7, 14.3%; CI95%: 0.4–57.9). Out of 7 GDPs, 3 resulted negative to the presence of *Sarcocystis* spp. DNA (Fig. 2).

Considering extralesional samples (*n* = 52), *Sarcocystis* DNA was detected in 46.2% of samples (24/52; CI95%: 32.2–60.5). *S. cruzi* was the most common species (30.8%; CI95%: 18.7–45.1), followed by *S. hominis* (15.4%; CI95%: 6.9–28.1), the unnamed *Sarcocystis* sp. (3.9%; CI95%: 0.5–13.2), *S. bovifelis* (1.9%; CI95%: 0.1–10.3) and *S. hirsuta* (1.9%; CI95%: 0.1–10.3) (Fig. 2). Mixed infections were observed in 5.8% (n = 3) of the extralesional samples, revealing the presence of two (*S. hominis* and *S. cruzi* or *S. bovifelis* and *S. cruzi*; n = 2) or three *Sarcocystis* spp. (*S. hominis*, *S. cruzi* and the unnamed *Sarcocystis* sp.; n = 1) at once.

The presence of *Sarcocystis* spp. DNA was significantly more frequent in intralesional samples than in extralesional samples (Fisher's exact test two-tailed, *P* < 0.0001). Considering the different *Sarcocysti*s spp., *S. bovifelis* and *S. hominis* were more frequently detected in intralesional (41.7% and 50%, respectively) than in extralesional samples (1.9% and 15.4%, respectively) (Fisher's exact test two-tailed, P < 0.0001 and *P* = 0.0003, respectively), while there was no significant difference between the presence of *S. cruzi*, the unnamed *Sarcocystis* sp. and *S. hirsuta* in intralesional (27.1%, 4.2% and 2.1%, respectively) and extralesional (30.8%, 3.9% and 1.9%, respectively) samples (Fisher's exact test two-tailed, *P* = 0.82, *P* > 0.9999, P > 0.9999, respectively). Taking into account the different lesion types (GDP and GFL), *Sarcocystis* spp. DNA was more frequently detected in GFL than in GDP (97.6% and 57.1%, respectively) (Fisher's exact test two-tailed, *P* = 0.007). The presence of *S. bovifelis* was more frequent in GFL than in GDP (48.8% and 0%, respectively) (Fisher's exact test two-tailed, *P* = 0.03), while there was no significant difference between the presence of *S. hominis*, *S. cruzi*, the unidentified *Sarcocystis* sp. and *S. hirsuta* in GFL and GDP.

Out of 100 muscle samples analyzed by multiplex-PCR, 4 samples collected from the skeletal muscles of 2 different BEM-condemned carcasses resulted in the presence of the *Sarcocystis* spp. amplicon (200 bp) with no species-specific fragments (previously mentioned as the unnamed *Sarcocystis* sp.) and were therefore sequenced. The obtained sequences didn't match with any other named *Sarcocystis* spp. sequences in the NCBI NT database, whilst showing a sequence homology of 97.2–99.4% with the GenBank entries n. MW582306-MW582307, FN394498-FN394500 (152–177 bp), corresponding to unidentified *Sarcocystis* spp. recorded in 2021 in North-West Italy (Rubiola et al., 2021) and in 2013 in Belgium (Vangeel et al., 2013) in cattle carcasses. One of the two affected carcasses could successfully be retrieved.

### 3.2 Morphological and molecular characterization of a new *Sarcocystis* species in cattle

**Fig. 3:**
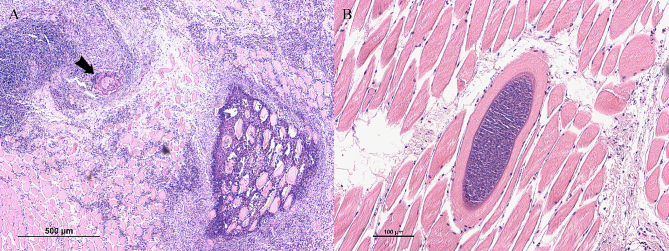
Histological sections of one of the two carcasses affected by bovine eosinophilic myositis (BEM) showing the presence of an unidentified *Sarcocystis* sp. (*S. sigmoideus* sp. nov.) A) Foci of eosinophilic and granulomatous myositis containing structures resembling degenerated cysts (arrow); B) Longitudinal section of an intact *S. sigmoideus* sp. nov. cyst detected outside BEM lesions.

**Fig. 4:**
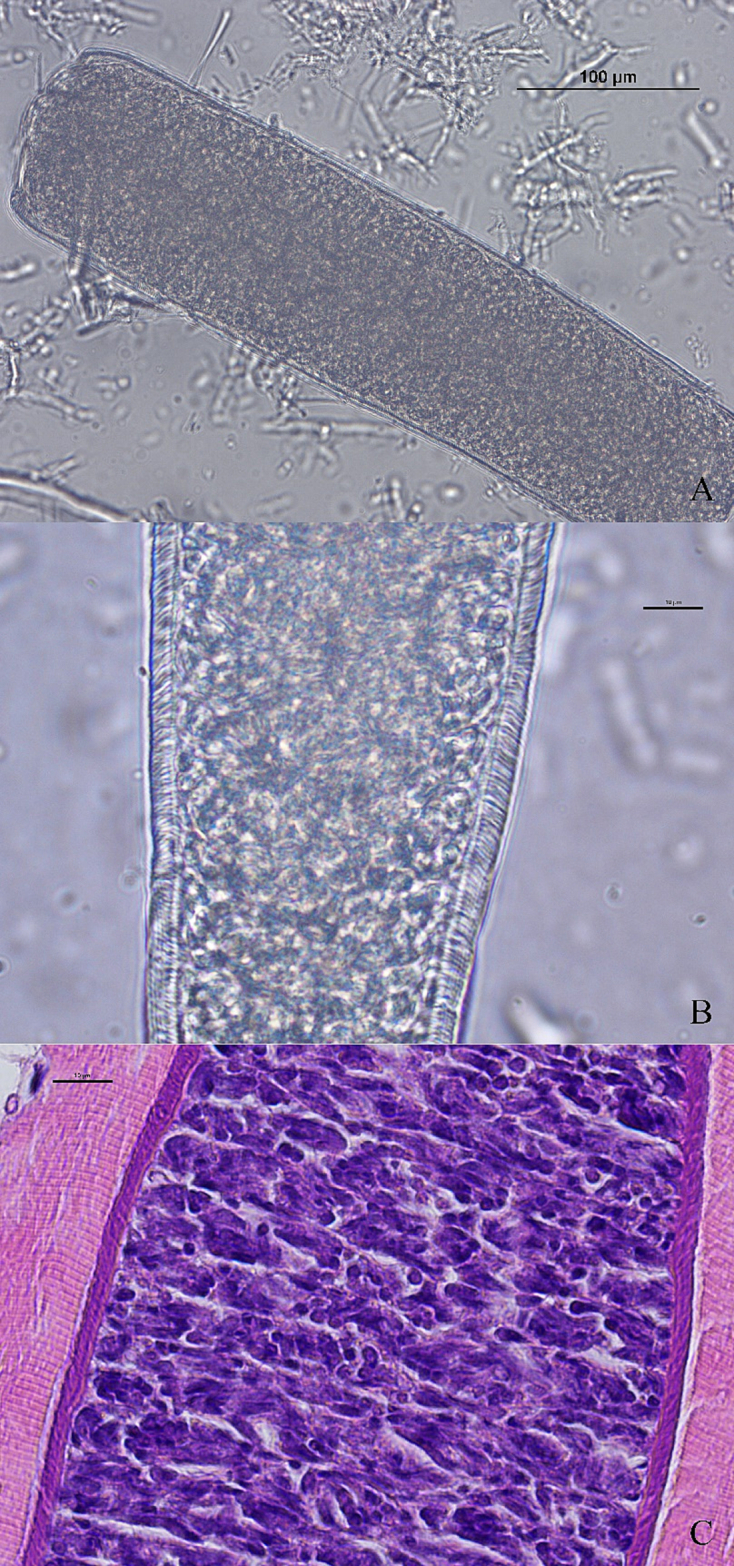
Morphology of sarcocysts of *Sarcocystis sigmoideus* sp. nov. from diaphragm muscles of a cattle (*Bos taurus*) affected by bovine eosinophilic myositis. Light microscope micrograph showing a sarcocyst portion by direct optical microscopy (A, B) and by microscopy of a H&E stained section (C) (scale bar A: 100 μm; scale bar B and C: 10 μm). As can be seen in both images, the cyst wall of *Sarcocystis sigmoideus* sp. nov. appears striated with highly packed, narrow protrusions, with a superficial dual refringence as outline. A): 200×. B) and C) images were obtained with the oil immersion objective (1000×).

Cysts isolation was performed on the diaphragm of one of the BEM-affected carcasses in which an unidentified *Sarcocystis* sp. was detected by multiplex-PCR. The corresponding heart of the same carcass evidenced only *S. cruzi* cysts. Muscle samples of the second carcass could not be recovered. The histopathology of this diaphragm revealed multiple foci of eosinophilic myositis containing structures resembling degenerated cysts (Fig. 3A, arrow). In surrounding unaffected muscle cells, thick-walled cysts containing narrow and densely packed protrusions were observed (Fig. 3B and Fig. 4), together with an intact thin-walled cyst (consistent with *S. cruzi*) (not shown).

**Supplementary Fig. S1:**
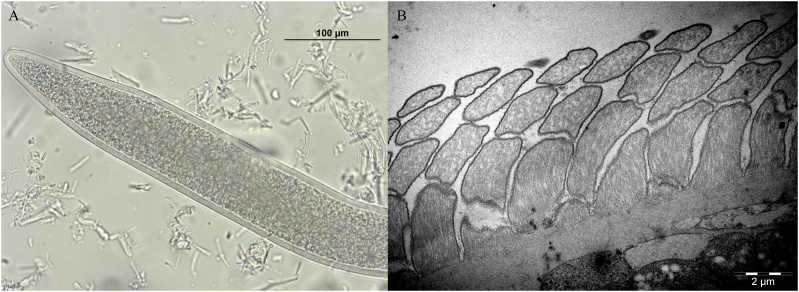
Supplementary Figure S1: Light microscope micrograph showing a thick-walled (7.5-9 μm) sarcocyst portion of *Sarcystis hominis* (A, magnification 200X). Transmission electron microscopy (TEM) image of the cyst wall of *S. hominis*, evidencing palisade-like protrusions containing microtubules (B). Cysts were recovered from a bovine diaphragm from Italy.

The direct microscopic examination of the muscle homogenate obtained applying a similar protocol to the one described by Moré et al. (Moré et al., 2013) revealed the presence of three morphologically distinct *Sarcocystis* spp. cysts, the majority of which were thick-walled. In total, 17 cysts or cyst portions were isolated and observed at optical microscopy. These included (i) one thin-walled cyst (cyst wall <1 μm), (ii) 10 thick-walled (3.7–5.4 μm) cyst portions with cyst walls showing a double outline of brightness and densely packed narrow protrusions (Fig. 4A and B), which were also observed in H&*E*-stained tissue sections (Fig. 4C), and (iii) six thick-walled (6.8–9.1 μm) cyst portions, whose cyst walls showed palisade-like protrusions (Suppl. Fig. 1A), compatible with *S. hominis* (molecularly confirmed) (Dubey et al., 2016; Gjerde, 2016a). The cysts wall measurements from all the cyst portions with a shared morphotype were performed with the Nikon NIS software. No complete thick-walled cysts were recovered for proper length measurements. Seven cyst portions observed at optical microscopy were recovered for TEM processing, together with further cysts collected from the muscle homogenate. An additional batch of 10 thick-walled cysts were recovered from diaphragm homogenate and processed by SEM.

**Fig. 5:**
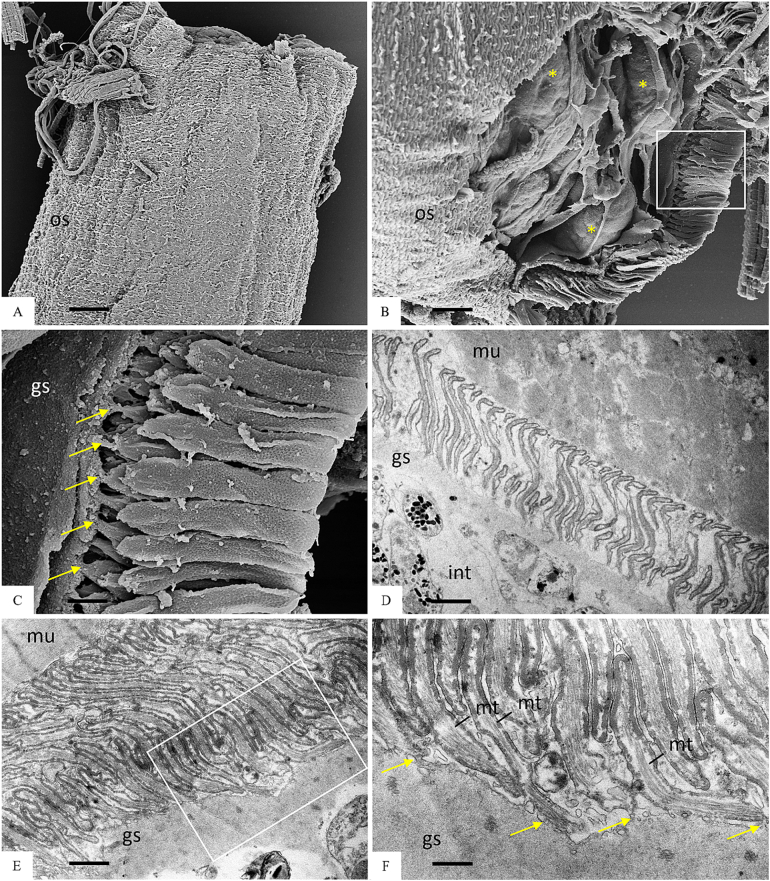
Ultrastructure of sarcocysts of *Sarcocystis sigmoideus* sp. nov. visualized by SEM (A–C) and TEM (D–F). A) Isolated cyst with muscle tissue removed, exposing the outer surface (os) with striated appearance. Fig. 5B) allows a view into the interior of the cyst, in which groups of bradyzoites, indicated by an asterisk, can be visualized. The boxed area is shown at higher magnification in C). The arrows indicate the narrow attachment points of the cyst wall protrusions to the ground substance layer (gs). D) and E) are TEM micrographs that reveal the sigmoid structure of the protrusions; the boxed area in E) is shown enlarged in F); mu = muscle tissue, int = cyst interior. Arrows in F) point towards attachment sites of the interior part of the sigmoid cyst wall structures to the cyst wall matrix; mt indicates the presence of cytoskeletal elements, most likely microtubules, extending to the villar tips. Bars in A) = 7.5 μm; B) = 5 μm; C) = 1.1. μm; D) = 2.7 μm; E) = 0.8 μm; F) = 0.4 μm.

**Supplementary Fig. S2:**
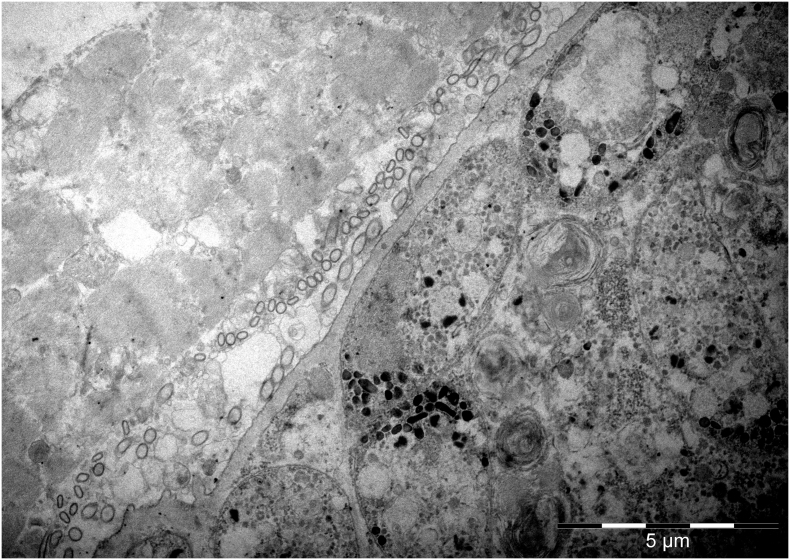
Transmission electron microscopy (TEM) image of a thin-walled cyst of *Sarcocystis cruzi* recovered from a bovine diaphragm, evidencing hair-like protrusions folder over the cyst surface and appearing as small circular structures in transversal sections.

By TEM, two different thick-wall types were observed: one resembling *S. hominis* with cylindrical to conical protrusions, tightly packed containing fine microtubules in the core (Suppl. Fig. 1B); the other showing thin, flattened and narrow protrusions around 5 μm long and 0.2–0.3 μm wide (longitudinal section side view), whose tips were bent at an angle, highly packed (up to three protrusions/μm) and undulating, showing an “S” form in a longitudinal section and side view. The base of the protrusions was narrower (100–150 nm) than the rest. The protrusions contained fine microtubules extending from base to tip; the microtubules did not extend into the thin ground substance layer (≤ 1 μm thick) (Fig. 5). The three sarcocysts having this TEM type were identified as the new *Sarcocystis* sp. and confirmed molecularly. Based on the cyst wall protrusions shape in a longitudinal section and side view, the name *Sarcocystis sigmoideus* sp. nov. is here proposed. A single thin-walled cyst was identified by TEM as *S. cruzi*: the cyst wall contained thin and long villar protrusions, folded over the cyst surface and appearing as small circular structures in transversal sections (Suppl. Fig. 2).

**Fig. 6:**
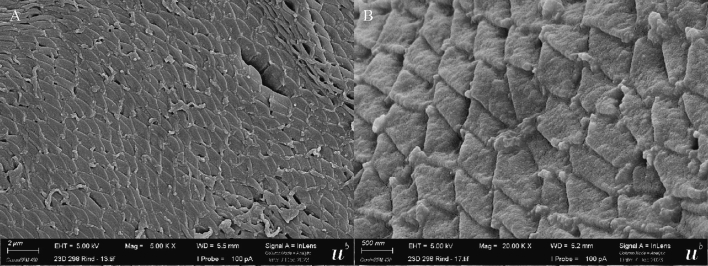
SEM micrograph showing the surface of a *S. sigmoideus* sp. nov. cyst. A) Densely packed and tightly overlapping cyst wall protrusion tips, giving the appearance of a tiled roof (scale bar: 2 μm). B) A higher magnification of the cyst surface showing the interconnected protrusion tips (scale bar: 0.5 μm).

**Supplementary Fig. S3:**
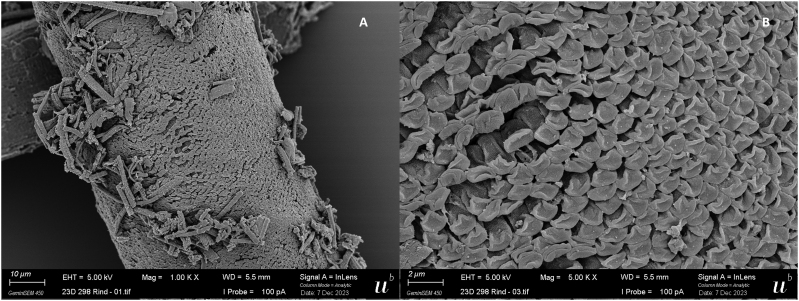
Scanning electron microscopy (SEM) images of a cyst of *Sarcocystis bovifelis* recovered from a bovine diaphragm. A) Overview of the cyst surface showing the protrusions tips which are folded and partially overlapping among them, giving the aspect of a tiled roof. B) Higher magnification of the cyst, showing flattened protrusions, with a tongue-like shape.

By SEM, also two different thick-wall types were observed: the first type showed tongue-like flattened protrusions with folded tips, partially overlapping among them, and giving the aspect of a tiled roof (Suppl. Fig. 3). These morphological characteristics are consistent with those of *S. bovifelis* as described by Gjerde (2016a). The other morphological type, corresponding to *S. sigmoideus* sp. nov., consisted of densely packed flattened protrusions, tightly overlapping, and apparently interconnected on the surface by transversal striations (Fig. 5 and Fig. 6). These protrusions were thin and broad (≤ 1 μm by ≤0.2 μm) with a narrower base (120–160 nm) and tips folding over and overlapping, resembling a tiled roof (Fig. 5 and Fig. 6). In a side view, the protrusions showed an undulating shape, consistent with the image obtained by TEM (Fig. 5).

DNA from 10 individual cysts (one thin-walled, four 6.8–9.1 μm thick-walled cysts and five 3.7–5.4 μm thick-walled cysts) was extracted and a partial mtDNA *cox1* gene fragment (∼ 1100 bp) of all the cysts was successfully amplified and sequenced. Out of 10 newly obtained sequences, the four *cox1* mtDNA sequences corresponding to the 6.8–9.1 μm thick-walled cysts (1005, 1014, 1017 and 1025 bp) showed 97.1–99.1% identity with *S. hominis* GenBank entries (accession no. MH021119, MK497840, MK497841, MK497842, MK497843) and <87.6% identity with other *Sarcocystis* spp. sequences available in GenBank; the *cox1* mtDNA fragment sequence (350 bp) corresponding to the thin-walled cyst showed a 98.6–99.7% identity with *S. cruzi* GenBank entries (accession no. MT796926-MT796945, MW507158-MW507159, MT796926, KT901078- KT901080, LC171859-LC171861) and <95.9% identity with other *Sarcocystis* spp. sequences available in GenBank; the five *cox1* sequences corresponding to the 3.7–5.4 μm thick cysts (1012, 1013, 1021, 1041 and 1041 bp) demonstrated 99.5–99.9% identity with an unidentified *Sarcocystis* sp. isolated in 2021 in bovine carcasses in Belgium (GenBank accession no. MW756133, MW756134, MW756135), and <83.4% identity with any other named *Sarcocystis* spp.

The complete 18S rDNA gene of one 6.8–9.1 μm thick-walled cyst and three 3.7–5.4 μm thick-walled cysts was successfully amplified and sequenced. The 1780 bp sequence of the thicker-walled cyst showed a 99.3–99.8% identity with *S. hominis* GenBank entries (accession no. JX679470, JX679471, KF954731, MT792481) and <97.3% identity with other *Sarcocystis* spp. sequences available in GenBank. The three complete 18S rDNA sequences of the 3.7–5.4 μm thick-walled cysts (1817, 1835 and 1844 bp) showed <94.6% identity with any *Sarcocystis* spp. sequences available in GenBank, while confirming a 98.7–99.4% identity with three small 18S rDNA fragments (152–177 bp) belonging to undescribed *Sarcocystis* spp. detected in cattle carcasses affected by eosinophilic myositis in 2013 in Belgium (Vangeel et al., 2013) and in 2021 in Italy (Rubiola et al., 2021) (accession no. MW582306, FN394498, FN394500).

As mentioned above, additional muscle samples of the second carcass affected by the unnamed *Sarcocystis* sp. could not be recovered; the DNA sample extracted from muscle was therefore directly subjected to the molecular characterization of the partial *cox1* gene and the complete 18S rDNA, as previously described. The obtained 1002 bp *cox1* mtDNA sequence showed a 99.8% identity with the five *cox1* mtDNA sequences resulting from the 3.7–5.4 μm thick-walled cysts isolated in the first carcass, 99.3–99.7% identity with the undescribed *Sarcocystis* sp. isolated in bovine carcasses in Belgium (GenBank accession no. MW756133, MW756134, MW756135) and <83.2% identity with any other named *Sarcocystis* sp. The 18S rDNA sequence (1749 bp) showed a 99.1% identity with the three 18S rDNA sequences resulting from the first carcass and <94.4% identity with any *Sarcocystis* spp. sequences available in GenBank.

Four BEM lesions detected in the first carcass were dissected under the stereomicroscope, isolated from the surrounding muscle fibers, and subjected to partial sequencing of the *cox1* mtDNA. The partial amplification and sequencing of the *cox1* mtDNA gene revealed the presence of *Sarcocystis* spp. DNA in each excised lesion. Sanger sequencing of three out of four lesions resulted in good quality sequences. The first BEM lesion resulted in a 1010 bp sequence showing 98.3–99.1% identity with *S. hominis* GenBank entries (accession no. MH021119, MK497840, MK497841, MK497842, MK497843) and <87.6% identity with other *Sarcocystis* spp. sequences available in GenBank. The second BEM lesion resulted in a 1014 bp sequence with a 98.8–100% identity with *S. bovifelis* GenBank entries (KT900961-KT900964, KT900966-KT900972, KT900974, KT900976, KT900980, KT900982, KT900990, KT900992, KT900994-KT900997, MT796903-MT796919, MT796920-MT796925, MK962348-MK962348, KC209690, KC209693-KC209696, OK041347-OK041350, OP235319) and <94.4% identity with other *Sarcocystis* spp. sequences available in GenBank. The third BEM lesion generated a 1048 bp sequence showing a 99.8–100% identity with the six *cox1* mtDNA sequences resulting from the 3.7–5.4 μm thick isolated cysts and from the second carcass, 99.5–99.9% identity with the undescribed *Sarcocystis* sp. from Belgium previously mentioned (GenBank accession no. MW756133, MW756134, MW756135) and <83.4% identity with any other named *Sarcocystis* sp.

**Fig. 7:**
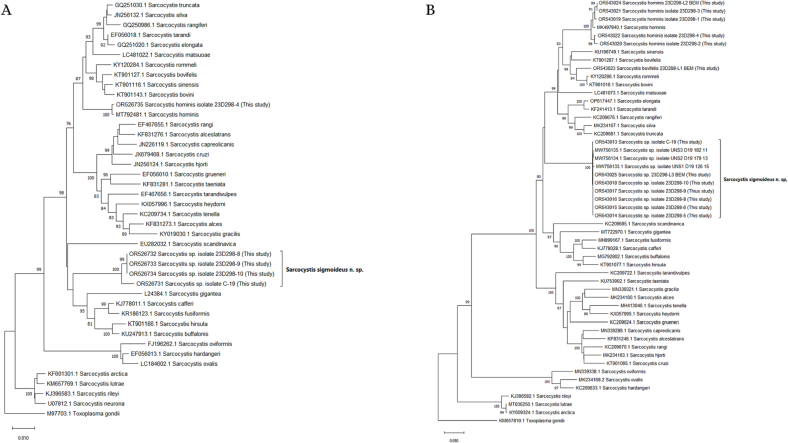
Neighbour joining phylogenetic trees based on the alignment of 42 near complete 18S rDNA sequences of 38 taxa, including 5 sequences generated in the present study (A), and 52 partial *cox1* mtDNA sequences of 37 taxa, including 13 sequences generated in the present study (B). Evolutionary distances were computed according to the Kimura two-parameter model. Branch consensus support is expressed as percentage from 1000 bootstraps and is reported next to the branches. Branch support values lower than 75% were not included. Trees were rooted on *Toxoplasma gondii*. GenBank accession numbers are included in the branches.

The phylogenetic analysis inferred from the 18S rDNA sequences positioned all the generated unidentified *Sarcocystis* sp. sequences together in a branch, including the sequence obtained from the first carcass (3 single-cysts sequences) and the sequence obtained from the second carcass (direct sequencing of the muscle DNA). Within the same clade, sequences from *S. buffalonis, S. hirsuta, S. fusiformis, S. cafferi*, *S. gigantea* and *S. scandinavica* were included*.* The clade formed a sister group to a major clade including different *Sarcocystis* spp. using ruminants (Bovidae and Cervidae) as intermediate hosts (*S. bovifelis, S. bovini, S. rommeli, S. sinensis, S. hominis, S. heydorni, S. cruzi, S. taeniata, S. matsuoae, S. tarandi, S. elongata, S. silva, S. rangiferi, S. truncate, S. rangi, S. alceslatrans, S. capreolicanis, S. hjorti, S. grueneri, S. tarandivulpes, S. tenella, S. alces* and *S. gracilis*)(Fig. 7A). The phylogenetic analysis based on partial *cox1* sequences also placed all the generated unidentified *Sarcocystis* sp. sequences together in a branch, including the sequence obtained from the first carcass (5 single-cysts and one BEM lesion sequences) and the sequence obtained from the second carcass (direct sequencing of the muscle DNA). The same branch included the undescribed *Sarcocystis* sp. isolated in bovine carcasses in Belgium (Zeng et al., 2021). Within the major sister clade, several *Sarcocystis* spp. using cervids (*S. silva, S. truncata, S. rangiferi, S. elongata, S. tarandi, S. matsuoae*) or bovids (*S. hominis, S. bovini, S. bovifelis, S. rommeli, S. sinensis*) as intermediate hosts were included (Fig. 7B).

All the sequences generated in the present study have been uploaded in the GenBank database with Accession Numbers OR543013-OR543025 (mtDNA *cox1* gene) and OR526731-OR526735 (18S rDNA gene).

#### 3.2.1 Taxonomic summary of *Sarcocystis sigmoideus* sp. nov.

Intermediate host: cattle (*Bos taurus*). Localization: sarcocysts and DNA were detected in diaphragm muscle.

Definitive host: unknown.

Distribution: Italy (Asti and Turin Prov., North-West Italy); Belgium. Prevalence appears to be relatively low, ranging from 1% to 7.7% (1/97 cattle in Belgium in 2013, 5/200 cattle in Belgium in 2021, 2/113 cattle in Italy in 2021 and 2/26 cattle in the current investigation).

Sarcocysts morphology: by light microscopy, sarcocysts were thick-walled (3.7–5.4 μm). At 200× magnification, the cyst wall appeared smooth and showed a double outline of brightness, with highly packed narrow protrusions barely visible; at 1000× magnification, densely packed narrow and bent protrusions were observed. By transmission electron microscopy, the cyst wall showed thin, flattened, and narrow protrusions around 5 μm long and 0.2–0.3 μm wide (longitudinal section side view), whose tips were bent at an angle. The protrusions were highly packed (up to three protrusions/μm) and undulating, showing an “S” form in a longitudinal section and side view. The base of the protrusions was narrower than the rest (100–150 nm). The protrusions contained fine microtubules extending from base to tip; the protrusions did not extend into the thin ground substance layer (≤ 1 μm thick).

By SEM, the cyst wall contained densely packed flattened protrusions, tightly overlapping, and apparently interconnected in the surface by transversal striations. The protrusions were thin and broad (≤ 1 μm by ≤0.2 μm) with a narrower base (120-160 nm) and tips folding over and overlapping with the interconnected protrusions. In a side view, the protrusions showed an undulating shape.

Molecular characteristics: 18S rDNA gene sequences (Accession numbers OR526731-OR526734) and cytochrome oxidase subunit I gene (*cox1*) sequences are available in GenBank (Accession numbers OR543013-OR543018; OR543025).

Deposited material: sequences of the *cox1* mtDNA and the 18S rDNA genes have been deposited in GenBank under the above-mentioned accession numbers. Genomic DNA of the examined isolates is being kept at the Department of Veterinary Sciences of the University of Turin (Turin, Italy) and at the Institute of Parasitology, Bern University (Bern, Switzerland). Histology slides of diaphragm muscle containing a *S. sigmoideus* sp. nov. cyst (Fig. 3B and Fig. 4B) were deposited at the Natural History Museum of Geneva (MHNG), Switzerland (registration number MHNG-INVE-0154235).

Etymology: The Ancient Greek word *“sigmoid”* (s-shaped) was used for the species name, with reference to the s-shaped protrusions observed in the cyst wall in a longitudinal section and side view.

## 4 Discussion

Bovine eosinophilic myositis is an inflammatory myopathy affecting striated muscles of cattle, which leads to carcass condemnation or discard of the affected parts and consequent economic losses for farmers. The role of *Sarcocystis* spp. in the onset of this pathology needs still to be clarified, but a number of studies have associated different *Sarcocystis* spp., producing both thin- and thick-walled cysts, with the presence of BEM lesions. This association was also reported in other animal species, as recently for red-deer (*Cervus elaphus*) (Basso et al., 2020). Herein, we investigated the presence of different *Sarcocystis* spp. inside and outside BEM lesions in condemned carcasses affected by eosinophilic myositis, and we reported the description of a new *Sarcocystis* species, *S. sigmoideus* sp. nov., based on light microscope, TEM, SEM, 18S rDNA, and the *cox1* mtDNA analysis.

In the present study, data related to BEM-condemnation between January 2014 and December 2019 in a big slaughterhouse of Northern Italy showed a BEM prevalence of 0.017%. Available data in Europe shows a lower prevalence, ranging from 0.002% in France to 0.003% in Belgium (Fortier et al., 1993); on the other hand, more data related to BEM-condemned carcasses are available for the USA, showing a higher prevalence ranging from 0.006 to 0.03%, with an exceptionally higher condemnation rate reported in 1986 for the western USA (5%) (Bradley and Taylor, 1993; Jensen et al., 1986; Fortier et al., 1993). Nevertheless, all the above-mentioned studies have been conducted three, four or more decades ago, while recent data are missing. To the best of our knowledge, this is the first report estimating BEM prevalence in Italy, as grossly classified by official veterinarians; studies investigating BEM prevalence in other countries would be needed in order to update the scarce and outdated reports actually available.

The current study examined 26 carcasses condemned with BEM-type lesions, most of which showed the presence of GFL (*n* = 41 from 22 carcasses), with a few more lesions categorized as GDP (*n* = 7 samples from 5 carcasses). All but one of the carcasses resulted positive to the presence of at least one *Sarcocystis* spp. Noteworthy, the lesions examined by *Taenia* spp. PCR resulted negative, ruling out the potential involvement of cysticercosis in the occurrence of lesions. The presence of *Sarcocystis* spp. DNA was significantly more frequent in intralesional samples than in extralesional samples (91.7% and 46.2%, respectively), thereby supporting the association of *Sarcocystis* spp. with eosinophilic myositis lesions. While *S. hominis*, *S. bovifelis*, *S. cruzi*, *S. hirsuta* and the newly named *S. sigmoideus* sp. nov. were detected in intralesional tissues, the presence of *S. hominis* and *S. bovifelis* was significantly more frequent in intralesional than in extralesional tissues; no significant difference between the presence of *S. cruzi*, the newly named *S. sigmoideus* sp. nov. and *S. hirsuta* in intralesional and extralesional samples was recorded. Taking into account the lesion categories, the presence of *Sarcocystis* spp. DNA was significantly more frequent in GFL than in GDP; among the different *Sarcocystis* spp. detected, only the presence of *S. bovifelis* DNA differed significantly, being more frequent in GFL.

These results are in accordance with a previous study conducted by Rubiola et al. in 2021 (Rubiola et al., 2021), which highlighted a significantly higher prevalence of *S. bovifelis* and *S. hominis* in carcasses affected by eosinophilic myositis compared to unaffected carcasses, although the difference between species detected in intra and extralesional tissues was not investigated. A possible major role of thick-walled *Sarcocystis* spp. in BEM etiology has been reported as well in 2013 by Vangeel et al. (Vangeel et al., 2013), whose analysis of BEM lesions resulted in the presence of intralesional *S. hominis* cysts in more than 80% of samples. As at that time the old classification including only *S. hominis*, *S. hirsuta* and *S. cruzi* was in force, sarcocysts identified as *S. hominis* by Vangeel et al. might have included other more recently named thick-walled *Sarcocystis* spp., such as *S. bovifelis* or *S. bovini*. Likewise, the presence of *S. hominis* cysts or other thick-walled sarcocysts in BEM lesions has been reported by Jensen et al. in 1986 in the USA (Jensen et al., 1986), by Gajadhar et al. in 1987 in North America (Gajadhar et al., 1987) and in 2023 in a case report by Dini et al. in Italy (Dini et al., 2023). Nevertheless, BEM has also been found in association with thin-walled cysts in other studies (Gajadhar and Marquardt, 1992; Dubey and Rosenthal, 2023), and as identifying *Sarcocystis* spp. inside BEM lesions might be complicated by the co-occurrence of more species and by the degeneration of the sarcocysts, further investigations applying simultaneously morphological and molecular methods to isolated lesions are required to unveil the enigma. In this context, the approach we used on the muscle tissues of the carcass affected by *S. sigmoideus* sp. nov., that is the individual isolation and dissection of BEM lesions under a stereomicroscope, with the removal of the surrounding muscle fibers and the molecular characterization of the lesions, has allowed us to identify the presence of *S. bovifelis*, *S. hominis* and *S. sigmoideus* sp. nov. in three different BEM core lesions collected from the same carcass. This finding further supports the assumption that thick-walled cysts may play a role in the etiology of an eosinophilic response in cattle.

The current investigation reports the detection and the morphological and molecular characterization of the previously undescribed *Sarcocystis sigmoideus* sp. nov. in the muscles of two cattle affected by eosinophilic myositis from Italy. The sarcocysts observed were microscopic and thick-walled, with highly packed narrow protrusions. From the diaphragm muscle sample which revealed the presence of *S. sigmoideus* sp. nov., *S. hominis* and *S. cruzi* cysts were also isolated and differentiated from the sarcocysts of *S. sigmoideus* sp. nov. under light microscopy, TEM and SEM due to the different width of the cyst wall (thin for *S. cruzi*, about 3.7–5.4 μm thick for *S. sigmoideus* sp. nov., and approx. 7–9 μm thick for *S. hominis*). The TEM and SEM studies allowed us to identify a new characteristic cyst wall ultrastructure for *S. sigmoideus* sp. nov. The protrusions clearly differed from the palisade-like cylindrical ones of *S. hominis* or the conical villar protrusions containing electron-dense granules of *S. hirsuta* (Dubey et al., 2016; Dubey, 2022; Gjerde, 2014a). The only other species with similar morphological features, showing tongue-like flattened protrusions with a thin ground substance layer is *S. bovifelis* (Gjerde, 2016a). In both species, the overview of the cyst surface resembles a tiled roof, but the protrusions are more densely packed in *S. sigmoideus*. The bases of the protrusions of *S. sigmoideous* sp. nov. are narrower than the ones of *S. bovifelis* (whose villar protrusions have broad bases that taper distally) and do not have vesicle or vesicle-like electron-lucent structures (Dubey, 2022; Gjerde, 2016a). Additionally, *S. sigmoideus* cysts have superficial striations (transversal to the cyst length), which are absent in *S. bovifelis*. These superficial striations along with the highly dense packing of flattened and undulating protrusions could be responsible for the dual brightness or refringence and the difficulties to reach a proper focus of the wall structure when observed by optical microscopy. Although these morphological features might be of help when performing the direct microscopic examination and electron microscopy differentiation, the molecular characterization of the *cox1* mtDNA gene and of the 18S rDNA gene reinforced and clearly separated phylogenetically *S. sigmoideus* sp. nov. from all other *Sarcocystis* spp. using cattle as intermediate hosts (Fig. 7). Based on the position of *S. sigmoideus* sp. nov. in the phylogenetic tree inferred using the 18S rDNA gene, which includes *S. sigmoideus* sp. nov. in a major cluster together with S. *buffalonis, S. hirsuta, S. fusiformis, S. cafferi, S. gigantea* and *S. scandinavica*, felids might be hypothesized as definitive hosts using this gene fragment phylogeny. On the other hand, the *S. sigmoideus* sp. nov. *cox1* mtDNA gene fragment sequences were positioned in a separate branch, and in a sister clade containing species using ruminants as intermediate hosts. Nevertheless, further research targeting different possible definitive hosts is needed to disclose the life cycle of this species.

Notably, exploring the literature including reports of unidentified and unnamed *Sarcocystis* spp. recorded in cattle, the molecular characterization of both the 18S gene and the mtDNA *cox1* gene has allowed us to retrospectively identify *S. sigmoideus* sp. nov. in a cattle carcass analyzed by Vangeel et al. in 2013 in Belgium (Vangeel et al., 2013), in two bovine carcasses analyzed by Rubiola et al. in 2021 in Italy (Rubiola et al., 2021) and in five carcasses analyzed by Zeng et al. in 2021 in Belgium (Zeng et al., 2021). The morphological description of an unnamed *Sarcocystis hominis*-like species reported by Domenis et al. in 2011 in Italy (Domenis et al., 2011), on the other hand, could more likely be attributable to *S. bovifelis*, despite this hypothesis cannot be confirmed, due to the low quality of the only publicly available sequence (Gjerde, 2016a). Nevertheless, since in our study and the others commented here, some samples with *S. sigmoideus* sp. nov. were also harboring *S. hominis* cysts, the potential zoonotic role of *S. sigmoideus* sp. nov. cannot be excluded and encourages further research.

Although *S. sigmoideus* sp. nov. was detected in 2 out of 26 cattle, it is impossible to estimate the actual prevalence, as all the sampled cattle carcasses were affected by BEM and the multiplex-PCR protocol applied didn't include the above-mentioned new species among the targeted species. Nevertheless, considering the present study and the studies performed by Vangeel et al. (2013), Zeng et al. (2021), and Rubiola et al. (2021), which detected the same novel species based on the molecular characterization here provided, the overall prevalence of *S. sigmoideus* sp. nov. in cattle appears to be quite low, ranging from 1% to 7,7% (1/97 cattle in Belgium in 2013, 5/200 cattle in Belgium in 2021, 2/113 cattle in Italy in 2021 and 2/26 cattle in the current investigation). Further studies targeting *S. sigmoideus* sp. nov. together with the other named bovine *Sarcocystis* spp. are required to uncover the real prevalence of *S. sigmoideus* sp. nov. in cattle and the possible association with eosinophilic myositis.

## 5 Conclusions

In conclusion, the current study contributes to our understanding of the importance of different *Sarcocystis* spp. in the BEM pathogenesis. The results emphasize the association of *S. hominis* and *S. bovifelis* with eosinophilic myositis and present the description of *S. sigmoideus* sp. nov.

*S. sigmoideus* sp. nov. is therefore the eighth named *Sarcocystis* spp. using cattle as intermediate hosts, together with *S. hominis*, *S. cruzi, S. hirsuta, S. heydorni, S. bovifelis, S. bovini* and *S. rommeli*.

The following are the supplementary data related to this article.

## Declaration of Competing Interest

The authors declare that they have no known competing financial interests or personal relationships that could have appeared to influence the work reported in this paper.

## Data Availability

The datasets generated in the present study are available in GenBank database with accession numbers OR526731-OR526735 and OR543013-OR543025.
